# Reducing the dose delivered to major intracranial blood vessels in brain radiotherapy using spot‐scanning proton arc therapy

**DOI:** 10.1002/acm2.70780

**Published:** 2026-09-09

**Authors:** Peilin Liu, Sebastian Tattenberg, Xiaoda Cong, Peter Y. Chen, Xuanfeng Ding, Abdelkhalek Hammi

**Affiliations:** ^1^ Department of Radiation Oncology William Beaumont University Hospital, Corewell Health Royal Oak Michigan USA; ^2^ Medical Physics, School of Medicine Wayne State University Detroit Michigan USA; ^3^ Laurentian University Sudbury Ontario Canada; ^4^ TRIUMF Vancouver British Columbia Canada; ^5^ Technical University of Dortmund Dortmund Germany; ^6^ Corewellhealth William Beaumont University Hospital Royal Oak USA

**Keywords:** blood dose, glioblastoma, lymphopenia, proton arc therapy, proton therapy

## Abstract

**Background:**

Glioblastoma (GBM) patients have poor clinical prognoses, which may partially be explainable by correlations between radiation‐induced killing of circulating blood cells (CBCs) and worse clinical outcomes. Proton radiotherapy is currently delivered via intensity‐modulated proton therapy (IMPT), for which the target is irradiated from a few different angles. Spot‐scanning proton arc therapy (SPArc), for which the target is irradiated from arc trajectories, is associated with dosimetric benefits, and the first SPArc treatments of head‐and‐neck cancer patients have now been reported. However, since SPArc increases the low‐dose bath, concerns have been raised about potentially higher killing of CBC.

**Purpose:**

This study aims to evaluate and compare blood vessel dose and dose‐averaged linear energy transfer (LET_d_) distributions between IMPT and SPArc, with and without blood vessel sparing, in patients with GBM.

**Methods:**

For 10 GBM patients, patient‐specific cerebrovasculature models were developed, and IMPT and SPArc treatment plans were created according to current clinical standards (IMPT_Conv_ and SPArc_Conv_) and with sparing of delineated blood vessels (IMPT_Sparing_ and SPArc_Sparing_) for every patient. Dose and LET_d_ distributions were then compared across all treatment plans.

**Results:**

While treatment plans maintained comparable target coverage, blood vessel sparing significantly reduced the mean dose to delineated blood vessels, with values of 6.5 ± 2.9 Gy(RBE) for IMPT_Conv_, 5.3 ± 2.3 Gy(RBE) for IMPT_Sparing_, 5.6 ± 2.2 Gy(RBE) for SPArc_Conv_, and 3.8 ± 1.6 Gy(RBE) for SPArc_Sparing_. The dose within the 10% of blood vessel volume receiving the highest dose (D_10%_) was 29.5 ± 13.4 Gy(RBE) for IMPT_Conv_, 23.2 ± 11.0 Gy(RBE) for IMPT_Sparing_, 24.5 ± 12.6 Gy(RBE) for SPArc_Conv_, and 13.4 ± 8.7 Gy(RBE) for SPArc_Sparing_.

**Conclusions:**

Implementation of blood vessel sparing resulted in successful reductions in blood vessel dose metrics while preserving target coverage, and SPArc further reduced blood vessel dose metrics compared with IMPT, with the potential to reduce radiation‐induced lymphopenia and improve clinical outcomes.

## 1 INTRODUCTION

Patients with glioblastoma (GBM) have a median survival time of less than 17 months, and standard treatment for GBM typically includes surgery, radiotherapy, and chemotherapy.^1^, ^2^ However, radiotherapy results in the killing of circulating blood cells (CBCs) as they flow through cerebrovascular vessels within the radiation field. This can lead to radiation‐induced lymphopenia—a reduction in lymphocyte levels—which has been associated with poorer outcomes in GBM and other cancers.^3^, ^4^, ^5^, ^6^, ^7^ Minimizing radiation exposure to major blood and lymphatic vessels through which the CBC flow could help mitigate lymphopenia and thereby improve survival outcomes.^8^, ^9^ Compared to conventional photon‐based techniques, intensity‐modulated proton therapy (IMPT) offers a significant advantage by delivering highly conformal dose distributions with up to four fields, better sparing organs‐at‐risk (OARs) beyond the target volume compared to conventional photon‐based radiotherapy. Additionally, IMPT reduces the dose delivered to CBCs compared to conventional photon therapy.^10^ However, intrinsic in vivo proton range uncertainties necessitate robust treatment plan optimization. To mitigate the impact of range uncertainties and patient setup errors, beam direction constraints are often implemented; however, these constraints may inadvertently increase the irradiated volume.^11^, ^12^, ^13^

Proton arc therapy (PAT), which involves target irradiation using a partial or full arc of gantry angles and which includes spot‐scanning proton arc therapy (SPArc), is a growing area of interest and has recently seen its first clinical applications in head‐and‐neck cancer.^14^, ^15^, ^16^ PAT has been shown to be associated with considerable dosimetric advantages compared to both photon therapy and IMPT, including lower integral doses, OAR doses, normal tissue complication probabilities (NTCPs), and/or secondary cancer probabilities, including for pediatric brain cancer patients.^17^, ^18^

However, PAT simultaneously increases the low‐dose bath compared to the limited‐beam configurations of IMPT, raising concerns about additional radiation exposure especially to the CBC, which are among the most radiosensitive cells in the body.^19^ Given the risk of radiation‐induced lymphopenia in GBM patients and its aforementioned correlation with worse clinical outcomes, it is essential to evaluate the potential trade‐offs between the superior OAR sparing associated with PAT and the potential drawbacks of the accompanying increase in the low‐dose bath. In brain radiotherapy, depletion of CBC is directly impacted by the dose to major intracranial vessels, which depends on both the spatial relationship between the vessels and the target volume and the resulting dose distribution.^20^ This study aims to determine whether PAT can reduce the dose to large blood vessels, which contain the majority of CBCs flowing through the brain, compared to IMPT.

For this purpose, four different proton therapy treatment plans were generated for a cohort of ten GBM patients: conventional IMPT (IMPT_Conv_) and SPArc (SPArc_Conv_) treatment plans, and IMPT and SPArc treatment plans which included sparing of a patient‐specific major blood vessel structure during radiotherapy treatment plan optimization (IMPT_Sparing_ and SPArc_Sparing_). The radiation dose as well as dose‐averaged linear energy transfer (LET_d_) within the tumor, OARs, and delineated blood vessels were then determined and analyzed for every treatment plan to verify whether it may be possible to leverage the higher degrees‐of‐freedom associated with SPArc to improve sparing of circulating lymphocytes compared to IMPT, without an accompanying increase in LET (and therefore relative biological effectiveness (RBE)).

## 2 METHODS

### 2.1 Patient selection and OAR delineation

Ten GBM patients were randomly selected from the “Glioma Image Segmentation for Radiotherapy: RT targets, barriers to cancer spread, and organs at risk” study (GLIS‐RT).^21^ A brain blood vessel phantom was then used to mimic the large blood vessels in the brains of each of the considered GBM patients. This blood vessel phantom was based on previously‐developed cerebrovascular phantoms designed to explicitly simulate the spatio‐temporal flow patterns of blood particles during fractionated radiotherapy of brain cancers.^22^, ^23^

The vascular tree includes principal arterial and venous structures that are commonly present in the healthy human brain. Arterial structures include the internal carotid arteries, the circle of Willis, and the anterior and middle cerebral arteries. Venous drainage is represented by superficial veins and dural venous sinuses, dominated by the superior sagittal sinus, which represents the largest vessel in the brain and runs along the midline of the brain. It drains into the transverse and sigmoid sinuses and then into the internal jugular veins. The phantom also includes alternative venous drainage pathways, such as the cavernous sinus system to reflect the multi‐pathway nature of cerebral venous outflow.

**FIGURE 1:**
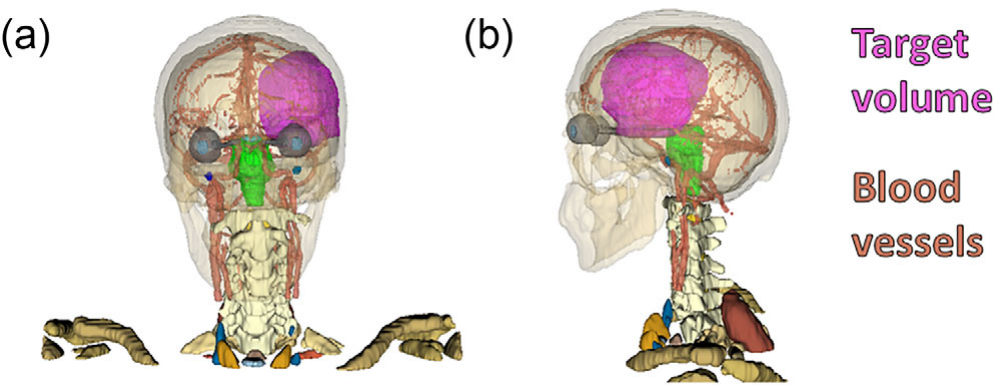
A (left) frontal and (right) side view of an example of (red) the delineated blood vessel structure and (pink) the target volume for one patient. For clarity, not all delineated structures are shown.

The purpose of using this standardized geometry was not to reproduce patient‐specific vascular anatomy, but to enable consistent, comparable estimation of dose to major vessels across patients and treatment techniques. To place the phantom in patient space, the atlas brain phantom was deformably registered to each patient's brain image using 3D Slicer, and the resulting deformation field was applied to propagate the vascular mask. Residual global misalignment was corrected with an additional forward affine refinement to improve axial alignment with the patient's brain contour. For quality control, maximum‐intensity projections of the transformed vascular mask were generated and overlaid with the cranial outline. The last two steps were repeated iteratively until major vessels were contained within the intracranial volume and the superior sagittal sinus aligned with the interhemispheric fissure (Figure 1).^20^ The registered blood vessel volume was reported in Table S1.

### 2.2 Treatment planning

Treatment plans were all generated in Raystation version 2023B (RaySearch Laboratories, Stockholm, Sweden). An IBA Proteus^®^ONE physics beam model (Ion Beam Applications, Louvain‐la‐Neuve, Belgium) was used for this study, with an energy range of 70–227 MeV and a corresponding spot size of 7.9–3.3 mm in air at isocenter.^24^ Four treatment plans with different treatment modalities and planning strategies were created for each patient.: an IMPT plan without sparing of major blood vessels (IMPT_Conv_), an IMPT plan with blood vessel sparing (IMPT_Sparing_), a SPArc plan without blood vessel sparing (SPArc_Conv_), and a SPArc plan with blood vessel sparing (SPArc_Sparing_). Which needs to be noticed is the blood vessel sparing plan with each kind of modalities also has the same objective functions compared to conventional IMPT and SPArc plan. Besides, the dose fall‐off functions have been applied to all blood vessel sparing plans with a relatively low weight (Table S2). Each IMPT plan consisted of 2 beams based on the tumor location and geometry, in accordance with clinical practice at our facility (Table S3). For the treatment plans for which blood vessels were considered during radiotherapy treatment planning, an additional dose fall‐off planning objective was added to the blood vessel structure. To enable a fair comparison of OAR sparing between planning techniques, all plans were normalized to ensure that 98% of the target volume received the prescription dose.

**FIGURE 2:**
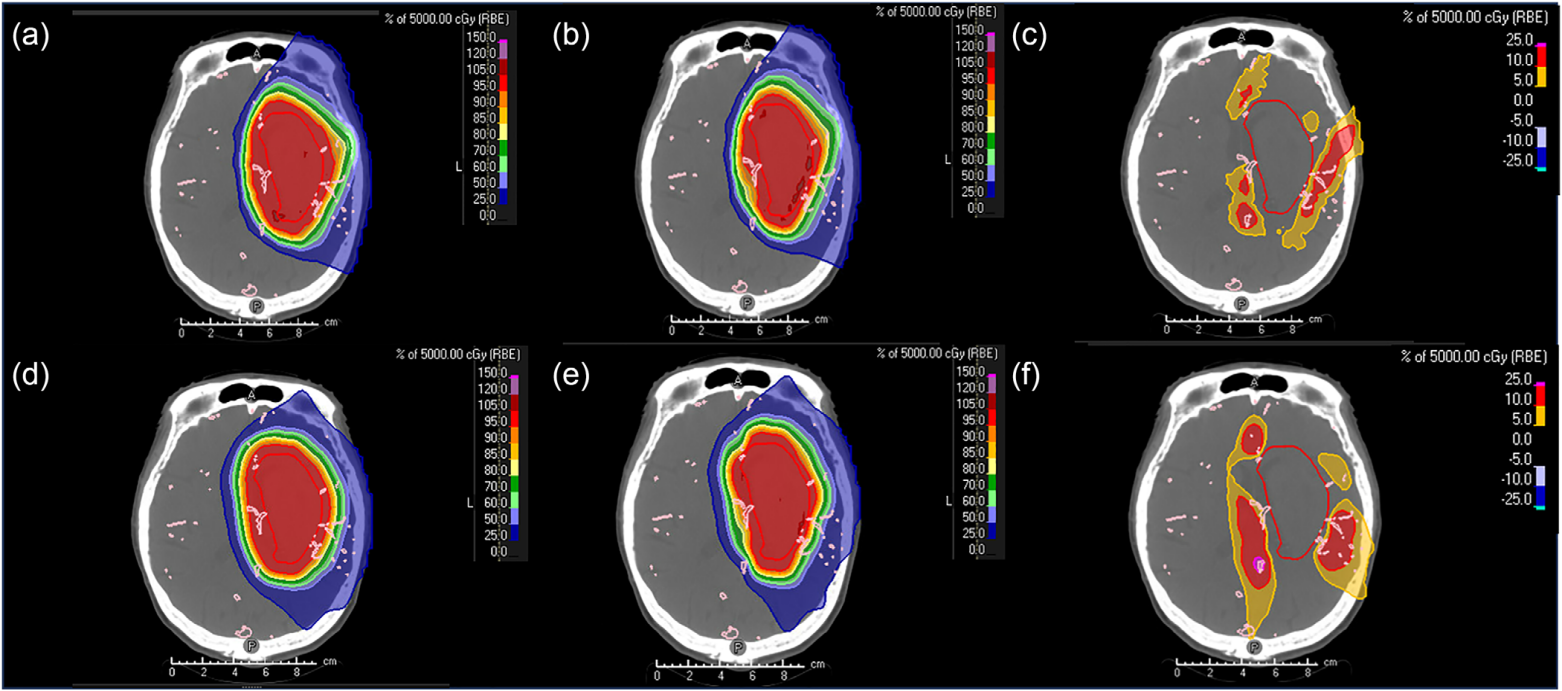
An example of dose distributions for one representative GBM patient illustrating the effect of treatment technique and blood vessel sparing strategy. Shown are (a) IMPT conventional (IMPT_Conv_), (b) IMPT with blood vessel sparing (IMPT_Sparing_), (c) the dose distribution difference between IMPT_Conv_ and IMPT_Sparing_ (IMPT_Conv_‐ IMPT_Sparing_), (d) SPArc conventional (SPArc_Conv_), (e) SPArc with blood vessel sparing (SPArc_Sparing_), and (f) the dose distribution difference between SPArc_Conv_ and SPArc_Sparing_ (SPArc_Conv_‐ SPArc_Sparing_), with the clinical target volume (red) and the delineated blood vessels (pink) interposed on the patient CT scan.

For SPArc, the treatment plans were created using an in‐house script in RayStation. The SPArc plan generation process involved several steps, such as control point re‐sampling, redistribution and filtering of energy layers, re‐sampling of energy layers, reduction in the number of spots, and multi‐field optimization.^14^ An initial control point sampling frequency of 2.5° was applied, and one patient‐specific partial arc was utilized. SPArc plans used the same OAR constraints and objectives and prescription doses as their IMPT counterparts. Robust optimization was performed with ± 3 mm setup uncertainty and ± 3.5% proton range uncertainty in accordance with our institutional clinical practice.^25^ The grid size of dose calcualtion was 1 mm × 1 mm × 1 mm. For a representative patient, all four treatment plans are shown in Figure 2.

### 2.3 Dosimetric parameter evaluation

A variety of dosimetric parameters were determined and compared across all treatment plans, target structures (Dose received by 98% of the volume, D98, conformity index, CI), OARs, and blood vessel structures (Dose received by 90% of the volume, D90, Dose received by 10% of the volume, D10).^26^ The CI of the target was defined as follows:

(1) $$\begin{array}{c} CI=\frac{TVI}{TIV}\;\# \end{array}$$

Here, TVI represents the reference isodose volume receiving at least 95% of the prescription dose in the target, and TIV represents the total isodose volume. The values of CI are between 0 and 1, with a CI closer to unity representing better dose conformity to the target.

### 2.4 Robustness evaluation

The dose distributions for all 21 error scenarios included during treatment planning were exported from Raystation and analyzed in Python. To ensure sufficient dose coverage of the target, 90% of the scenarios were required to satisfy the condition that 95% of the target volume received at least 95% of the prescription dose. In addition, even in the worst‐case scenario, at least 90% of the target volume was required to receive a dose of at least 90% of the prescription dose, in accordance with clinical practice.

### 2.5 LET distribution evaluation

In addition, Monte Carlo simulations were performed in RayStation in order to determine the dose‐averaged linear energy transfer scored in water (LET_d_), which was defined as follows^27^:

(2) $$\begin{array}{c} \;LE\;T_{d,wat}=\frac{\sum_{n=1}^{N}\sum_{s=1}^{s_{n}}\omega_{n}L_{sn,wat}\varepsilon_{sn}{\left(\frac{S}{\rho }\right)}_{med}^{wat}}{\sum_{n=1}^{N}\sum_{s=1}^{s_{n}}\omega_{n}\varepsilon_{sn}{\left(\frac{S}{\rho }\right)}_{med}^{wat}}\;\# \end{array}$$

Here, N is the total number of particles that deposit energy within the voxel in question, n denotes a single charged particle that traverses the voxel in $s_{n}$ steps, $\omega_{n}$ is the weight of particle n, and s denotes a specific step in the path of particle n through the voxel. 𝐿_𝑠𝑛, *wat*_ is the mean energy lost per unit path length traversed by the particle in water, $\varepsilon_{sn}$ is the electron energy lost by the particle in step s, and ${\left(\frac{S}{\rho }\right)}_{med}^{wat}$ is the mass stopping power of the particle in water divided by the mass stopping power of the particle in the medium. $LET_{d,wat}$ was computed and analyzed for all target volumes as well as the healthy brain tissue.

### 2.6 Statistical analysis

Version 9.0 of Prism (San Diego, CA, USA) as well as Python were used for data analysis. Given the limited cohort size (*n* = 10), paired comparisons were utilized two‐sided Wilcoxon signed‐rank tests. Four pre‐specified pairwise comparisons were performed for each dosimetric and LETd endpoint. To account for multiple comparisons, p‐values were adjusted using the Holm–Bonferroni method. In addition, effect sizes were quantified using the matched‐pairs rank‐biserial correlation, and Hodges–Lehmann median differences with 95% confidence intervals were reported. A two‐sided Holm‐adjusted *p* < 0.05 was considered statistically significant.

## 3 RESULTS

### 3.1 Treatment plan evaluation

In order to ensure that all four treatment plans created for every patient were associated with a comparable target coverage, Table 1 provides the minimum dose received by 98% of the target volume (D_98%_) and CI for all patients and treatment plans. For all four treatment approaches, the mean D_98%_ over all patients was 50.00 Gy(RBE) with standard deviations of 0.01 Gy(RBE) for IMPT_Conv_ and SPArc_Conv_ and 0.00 Gy(RBE) for IMPT_Sparing_ and SPArc_Sparing_ with no statistically significant differences (all *p* > 0.05). The conformity index values were 0.54 ± 0.06 (IMPT_Conv_), 0.60 ± 0.06 (IMPT_Sparing_), 0.55 ± 0.08 (SPArc_Conv_), and 0.58 ± 0.07 (SPArc_Sparing_). Wilcoxon signed‐rank testing yielded *p* = 0.008 for IMPT_Sparing_ versus IMPT_Conv_ and *p* = 0.059 for SPArc_Sparing_ versus SPArc_Conv_, while comparisons between IMPT_Conv_ versus SPArc_Conv_ and IMPT_Sparing_ versus SPArc_Sparing_ resulted in *p* = 0.516 and *p* = 0.516, respectively (Tables 1 and S4).

**TABLE 1:** The minimum dose received by 98% of the target volume (D_98%_) and CI for the intensity‐modulated proton therapy (IMPT) and SPArc treatment plans without sparing of delineated blood vessels (IMPT_Conv_ and SPArc_Conv_) and with sparing of delineated blood vessels (IMPT_Sparing_ and SPArc_Sparing_) during radiotherapy treatment planning.

	D_98%_ [Gy(RBE)]				CI			
Case number	IMPT_conv_	IMPT_Sparing_	SPArc_conv_	SPArc_Sparing_	IMPT_Conv_	IMPT_Sparing_	SPArc_Conv_	SPArc_Sparing_
1	50.00	50.00	50.00	50.00	0.56	0.66	0.70	0.66
2	50.02	50.00	50.02	50.00	0.62	0.67	0.60	0.65
3	50.00	50.00	50.00	50.00	0.52	0.53	0.49	0.52
4	50.00	50.00	50.00	50.00	0.50	0.55	0.53	0.58
5	50.00	50.00	50.00	50.00	0.51	0.61	0.54	0.56
6	50.00	50.00	50.00	50.00	0.54	0.59	0.52	0.56
7	50.00	50.00	50.00	50.00	0.49	0.57	0.55	0.60
8	50.00	50.00	50.00	50.00	0.66	0.69	0.66	0.68
9	50.00	50.00	50.00	50.00	0.45	0.50	0.45	0.47
10	50.00	50.00	50.00	50.00	0.54	0.58	0.50	0.55
Mean	50.00	50.00	50.00	50.00	0.54	0.60	0.55	0.58
Standard deviation	0.01	0.00	0.01	0.00	0.06	0.06	0.08	0.07

Normal brain and OAR dosimetric parameters are summarized in Table S5. Compared with IMPT_Conv_, IMPT_Sparing_ reduced normal brain V5Gy(RBE) from 32.76 ± 9.04% to 30.35 ± 8.98%, V10 Gy(RBE) from 27.95 ± 7.91% to 25.60 ± 7.71%, V20 Gy(RBE) from 21.58 ± 5.97% to 19.16 ± 5.43%, and V25 Gy(RBE) from 18.78 ± 5.13% to 16.37 ± 4.52% (all *p* = 0.0117). Similarly, compared with SPArc_Conv_, SPArc_Sparing_ reduced normal brain V5 Gy(RBE) from 34.08 ± 12.10% to 28.23 ± 10.56%, V10 Gy(RBE) from 25.71 ± 7.90% to 21.90 ± 7.18%, V20 Gy(RBE) from 18.53 ± 5.22% to 15.70 ± 4.65%, and V25 Gy(RBE) from 16.16 ± 4.47% to 13.56 ± 4.01% (all *p* = 0.0117). SPArc_Sparing_ also reduced brainstem D0.03cc from 38.12 ± 20.70 Gy(RBE) to 32.56 ± 20.47 Gy(RBE) (all *p* = 0.0234), right optic nerve D0.03cc from 13.16 ± 20.90 to 11.65 ± 20.51 Gy(RBE) (*p* = 0.0234), and left optic nerve D0.03cc from 15.76 ± 18.88 to 11.57 ± 16.35 Gy(RBE) (*p* = 0.0234).

### 3.2 Dose and LET_d_ of major blood vessels

**FIGURE 3:**
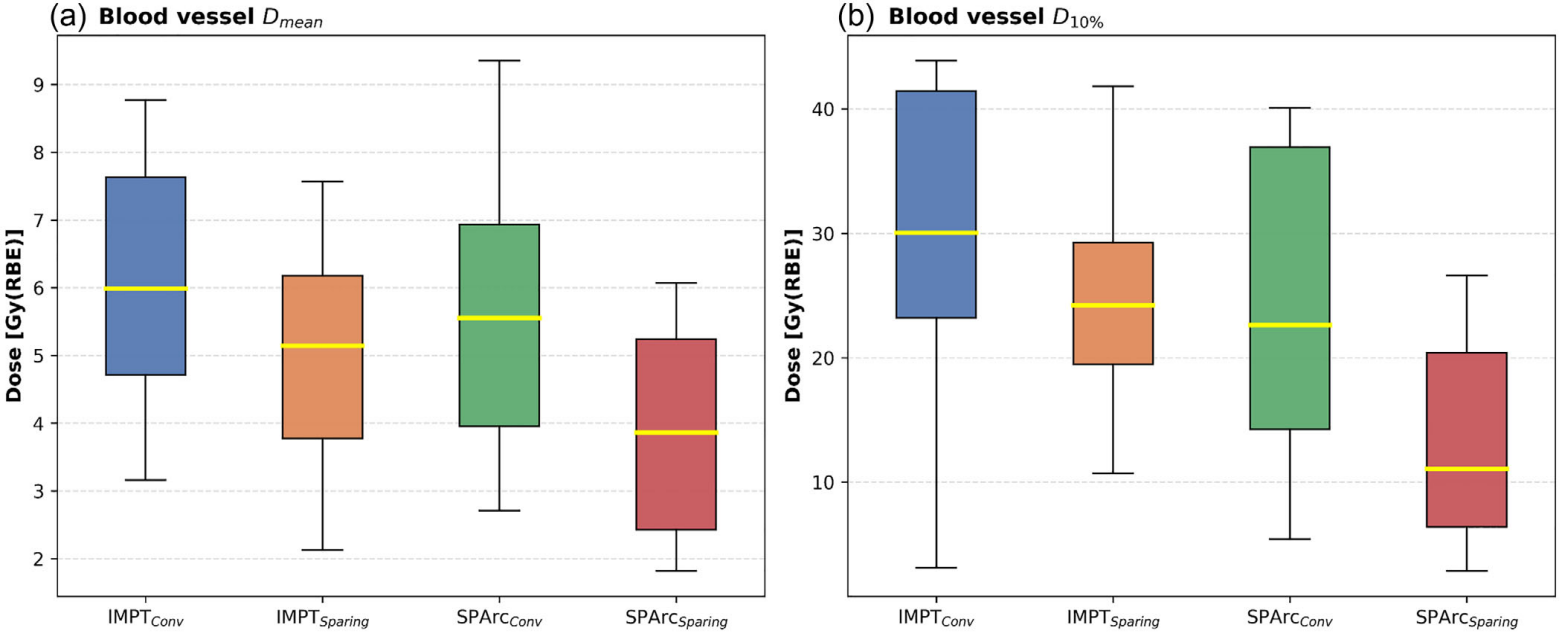
Distribution of blood vessel dose metrics (D_mean_ and D_10%_) for IMPT and SPArc treatment plans with (IMPT_Sparing_ and SPArc_Sparing_) and without blood vessel sparing (IMPT_Conv_ and SPArc_Conv_). In this figure, the yellow line represents the median value of each group.

For all four treatment modality and treatment planning approach combinations, the mean dose in the delineated blood vessels (D_mean_) as well as the dose received by the 10% of blood vessel volume receiving the highest dose (D_10%_) are shown in Table 2. The mean dose to the blood vessel structure was 6.53 ± 2.87 Gy(RBE) for IMPT_Conv_, 5.30 ± 2.27 Gy(RBE) for IMPT_Sparing_, 5.59 ± 2.17 Gy(RBE) for SPArc_Conv_, and 3.83 ± 1.61 Gy(RBE) for SPArc_Sparing_. Wilcoxon signed‐rank testing with Holm–Bonferroni correction showed statistically significant differences in blood vessel D_mean_ for IMPT_Conv_ compared to IMPT_Sparing_ (*p* = 0.008), SPArc_Conv_ compared to SPArc_Sparing_ (*p* = 0.008), IMPT_Conv_ compared to SPArc_Conv_ (*p* = 0.010), and IMPT_Sparing_ compared to SPArc_Sparing_ (*p* = 0.008). Values of D_10%_ were 29.52 ± 13.36 Gy(RBE) for IMPT_Conv_, 23.24 ± 11.03 Gy(RBE) for IMPT_Sparing_, 24.45 ± 12.59 Gy(RBE) for SPArc_Conv_, and 13.40 ± 8.70 Gy(RBE) for SPArc_Sparing_ (Tables 2 and S6). The dosimetric data are summarized in Figure 3.

**TABLE 2:** The mean dose to all delineated blood vessels (D_mean_) and the dose to the 10% of blood vessel volume receiving the highest dose (D**_10_**_%_) for the intensity‐modulated proton therapy (IMPT) and SPArc treatment plans without sparing of delineated blood vessels (IMPT_Conv_ and SPArc_Conv_) and with sparing of delineated blood vessels (IMPT_Sparing_ and SPArc_Sparing_) during radiotherapy treatment planning.

	D_mean_ [Gy(RBE)]				D_10%_ [Gy(RBE)]			
Case number	IMPT_conv_	IMPT_Sparing_	SPArc_conv_	SPArc _Sparing_	IMPT_Conv_	IMPT _Sparing_	SPArc_conv_	SPArc _Sparing_
1	3.63	2.13	2.71	1.82	43.87	28.01	35.20	20.30
2	8.77	7.57	8.16	6.07	40.31	29.68	40.08	23.72
3	3.16	2.97	3.32	2.41	3.12	2.72	5.43	2.87
4	5.30	4.80	3.99	2.49	22.59	19.45	13.02	5.70
5	4.52	3.43	3.94	1.97	15.65	10.69	11.81	3.70
6	7.26	6.38	6.10	4.58	29.15	24.37	20.97	12.37
7	5.45	4.82	5.52	4.06	25.06	19.53	17.96	9.78
8	12.89	9.85	9.35	5.46	42.65	32.03	37.49	20.44
9	7.76	5.57	7.21	5.79	41.82	41.81	38.28	26.62
10	6.53	5.47	5.59	3.66	30.97	24.06	24.29	8.52
Mean	6.53	5.30	5.59	3.83	29.52	23.24	24.45	13.40
Standard deviation	2.87	2.27	2.17	1.61	13.36	11.03	12.59	8.70

LET_d_ data for all patients and treatment plans is shown in Table 3. For the target, LET_d,2%_, values for IMPT_Conv_, IMPT_Sparing_, SPArc_Conv_, and SPArc_Sparing_ were 3.3 ± 0.3, 3.3 ± 0.3, 3.1 ± 0.2, and 3.3 ± 0.2 keV/µm, and the corresponding LET_d, mean_ values were 2.4 ± 0.2, 2.4 ± 0.2, 2.4 ± 0.1, and 2.4 ± 0.1 keV/µm, respectively. Statistical analysis with Holm–Bonferroni correction resulted in *p* = 0.375, *p* = 0.039, *p* = 0.500, and *p* = 0.500 for LET_d,2%_ and *p* = 0.750, *p* = 0.500, *p* = 1.000 and *p* = 1.000 for LET_d, mean_, corresponding to comparisons of IMPT_Conv_ versus IMPT_Sparing_, SPArc_Conv_ versus SPArc_Sparing_, IMPT_Conv_ versus SPArc_Conv_, and IMPT_Sparing_ versus SPArc_Sparing_, respectively. For delineated blood vessels, LET_d,2%_ values were 9.9 ± 1.9, 9.9 ± 1.9, 8.9 ± 1.3, and 9.2 ± 0.9 keV/µm, while LET_d, mean_ values were 2.9 ± 1.0, 2.8 ± 0.9, 2.7 ± 0.5, and 2.7 ± 0.6 keV/µm for IMPT_Conv_, IMPT_Sparing_, SPArc_Conv_, and SPArc_Sparing_, respectively. No statistically significant differences were observed for blood vessel LET_d_ parameters (*p* > 0.05 for all pairwise comparisons) (Tables 3 and S7).

**TABLE 3:** Mean LETd (LET_d, mean_) and LETd within 2% of the structure (LETd_,2%_) for the clinical target volume and the blood vessels for every patient. Values are shown for the intensity‐modulated proton therapy (IMPT) and Spot‐scanning proton arc therapy (SPArc) treatment plans without sparing of delineated blood vessels (IMPT_Conv_ and SPArc_Conv_) and with sparing of delineated blood vessels (IMPT_Sparing_ and SPArc_Sparing_) during radiotherapy treatment planning.

Case number	Clinical target volume								Blood vessels							
	LET_d,2%_ [keV/µm]				LET_d, mean_ [keV/µm]				LET_d,2%_ [keV/µm]				LET_d, mean_ [keV/µm]			
	IMPT_Conv_	IMPT_Sparing_	SPArc_Conv_	SPArc_Sparing_	IMPT_Conv_	IMPT_Sparing_	SPArc_Conv_	SPArc_Sparing_	IMPT_Conv_	IMPT_Sparing_	SPArc_Conv_	SPArc_Sparing_	IMPT_Conv_	IMPT_Sparing_	SPArc_Conv_	SPArc_Sparing_
1	3.3	3.4	3.3	3.5	2.3	2.4	2.4	2.5	10.1	10.4	9.5	9.5	3.7	3.6	3.0	2.6
2	3.4	3.4	3.0	3.1	2.5	2.6	2.5	2.5	10.6	10.0	9.1	10.4	2.0	2.0	2.8	2.8
3	3.4	3.4	3.4	3.3	2.6	2.6	2.5	2.5	7.7	8.0	9.6	9.3	1.0	1.0	1.6	1.5
4	2.5	2.5	3.2	3.5	2.0	2.0	2.3	2.4	6.0	6.3	10.1	10.4	3.1	3.2	3.4	3.5
5	3.3	3.5	3.1	3.4	2.4	2.5	2.5	2.5	10.5	10.7	7.3	8.2	3.4	3.3	2.4	2.6
6	3.7	3.8	3.0	3.1	2.6	2.6	2.4	2.5	11.7	12.1	7.6	8.0	2.7	2.8	2.9	2.9
7	3.3	3.3	3.0	3.2	2.4	2.4	2.3	2.4	12.5	12.9	7.8	8.9	4.6	3.9	2.1	2.3
8	3.2	3.2	2.9	3.1	2.2	2.2	2.2	2.2	9.7	9.1	7.2	8.0	3.4	3.4	3.2	3.5
9	3.0	3.1	3.0	3.1	2.2	2.2	2.3	2.3	9.0	9.0	10.1	10.2	3.3	3.3	3.1	3.1
10	3.5	3.5	3.2	3.4	2.6	2.6	2.5	2.5	10.9	10.9	10.9	9.0	1.9	1.8	2.6	2.5
Mean	3.3	3.3	3.1	3.3	2.4	2.4	2.4	2.4	9.9	9.9	8.9	9.2	2.9	2.8	2.7	2.7
Standard deviation	0.3	0.3	0.2	0.2	0.2	0.2	0.1	0.1	1.9	1.9	1.3	0.9	1.0	0.9	0.5	0.6

### 3.3 Robustness evaluation

**FIGURE 4:**
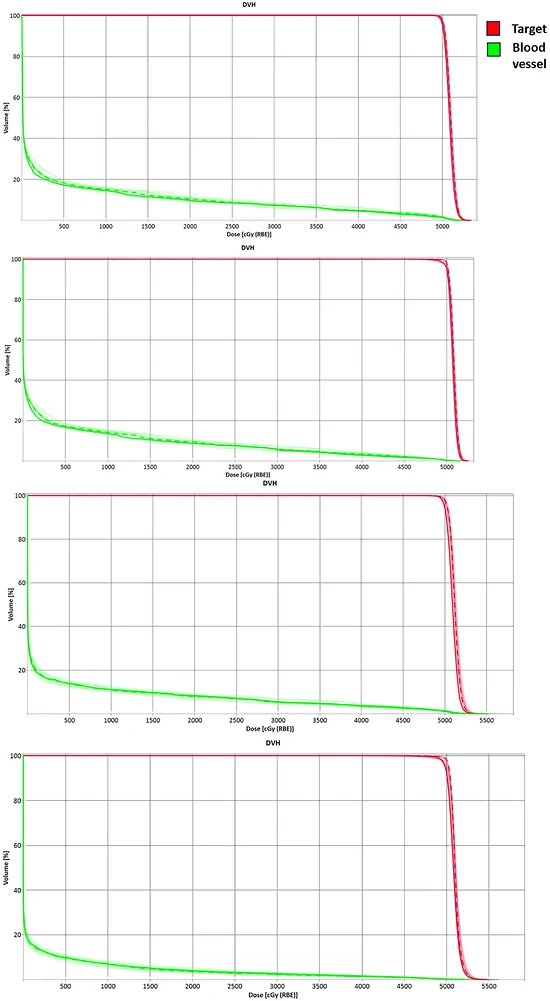
Robustness evaluation of dose distributions for a representative patient, showing (red) clinical target volume and (pink) blood vessel dose distributions for all scenarios included during treatment planning for (a) IMPT_Conv_, (b) IMPT_Sparing_, (c) SPArc_Conv_ and (d) SPArc_Sparing._ The solid line represents the nominal scenario, and the dashed lines represent the worst‐case scenarios.

Robustness evaluation demonstrated that all IMPT and SPArc treatment plans, with and without blood vessel sparing, fulfilled the clinical robustness criteria. Most importantly, each plan preserved the required target coverage in all 21 evaluated uncertainty scenarios encompassing setup and range perturbations. An example robustness evaluation plot is provided in Figure 4.

## 4 DISCUSSION

While all four treatment plans generated for every patient exhibited consistent target coverage and comparable LET_d_ distributions, blood vessel sparing during both IMPT and SPArc was able to significantly (*p* < 0.05) reduce the dose delivered to the delineated blood vessels. These differences remained significant after Holm–Bonferroni correction (all adjusted *p* ≤ 0.008) and were associated with large effect sizes (matched‐pairs rank‐biserial *r* = 0.89–1.00). At the same time, SPArc was associated with considerably lower blood vessel dose metrics compared to IMPT both with and without inclusion of the delineated blood vessels. This is in agreement with the literature, since it has been reported that PAT is able to considerably reduce OAR dose and/or integral doses compared to conventional radiotherapy techniques, including for pediatric brain cancer patients.^17^, ^18^ The observed reductions in dose to the delineated major intracranial vessel structure while maintaining target coverage may contribute to reducing radiation‐induced lymphopenia and potentially improving clinical outcomes.^3^, ^4^, ^5^

Although the vessel phantom used in this study captures the major cerebrovascular anatomy that is broadly consistent across healthy adults, it remains a simplified representation of the intracranial arterial and venous tree and cannot reproduce patient‐specific branching patterns or inter‐ and intra‐subject variability. Such variability can influence the dose delivered to flowing blood in a technique‐dependent manner and has been reported to introduce uncertainties of up to ∼18% in estimated irradiated blood volume, which may propagate to an uncertainty of ∼11% (1σ) in the predicted fraction of depleted CBC.^20^ Future work will therefore focus on extending the phantom with more vasculature detail.

While inter‐subject vascular variability will still affect the reported dose to blood vessel metrics, prior photon‐therapy studies suggest that arc‐based delivery (VMAT) is less sensitive to uncertainties arising from non‐patient‐specific vessel anatomy than fixed‐field IMRT. By the same rationale, SPArc is expected to be more robust than fixed‐beam IMPT to realistic variations in major‐vessel geometry because the dose is delivered from a broader range of angles, reducing dependence on any single vessel–beam configuration.

To reduce this uncertainty, future work should move toward patient‐specific cerebrovascular models derived from angiographic MRI. This would allow us to test, across anatomically diverse patients, whether SPArc consistently reduces dose to the cerebrovasculature relative to fixed‐beam IMPT. Although the phantom was registered to the patient's CT using the MRI‐to‐CT transformation matrix, the final adjustment of its orientation and position was assessed qualitatively. This is a limitation of the workflow, as it is user‐dependent and may be difficult to reproduce. Future studies should implement quantitative alignment criteria, such as alignment of the superior sagittal sinus with the interhemispheric fissure or Dice similarity metrics between superficial vessels and the cortical surface, to improve reproducibility. In order to confirm that the observed reductions in blood vessel doses translate into reductions in doses delivered to circulating lymphocytes, it will be necessary to perform full dynamic blood flow simulations (i.e., simulations which account for both blood flow as well as treatment delivery dynamics) of radiation dose delivery to circulating blood during radiotherapy treatment delivery. These simulations will be required to account for the vast majority of (rather than just the major delineated) blood vessels within the brain for realism, as a dynamic model solely capturing the major delineated blood vessels would be insufficient, as the current work already shows that the dose delivered to the aforementioned blood vessels can be significantly reduced. Such a limited dynamic model would therefore be biased towards confirming a resulting reduction in circulating lymphocyte doses rather than constituting a more independent verification. The dynamic model will furthermore be required to be capable of performing simulations with a particularly high temporal resolution, since accurate simulation of the delivery dynamics of PAT, due to the considerable number of gantry angles, and especially when considering dynamic treatment delivery during gantry rotation, is far from straightforward. Furthermore, treatment delivery time may influence the dose received by circulating lymphocytes. In the present study, the total beam delivery time, including energy layer switching time within each beam, beam switching time between beams, spot switching time, and spot drill time, SPArc plans required approximately 30% longer delivery times than IMPT plans (164.1 and 160.1 s for SPArc_Conv_ and SPArc_Sparing_ versus 127.5 and 126.0 s for IMPT_Conv_ and IMPT_Sparing_, respectively; Table S8). Assuming continuous blood circulation during treatment, longer delivery times may distribute the delivered dose across a larger volume of circulating blood, potentially further reducing the dose received by individual lymphocytes. While outside of the scope of this study, the expansion and testing of a dynamic blood flow model which can sufficiently satisfy all of the aforementioned requirements is now underway, and future work will focus on evaluation (and eventually also blood dose optimization) utilizing fully dynamic, high temporal resolution blood dose simulations.

A potential future direction is to incorporate NTCP models directly into the optimization process. Current planning strategies primarily rely on dose–volume constraints, which may not fully capture the biological effects of radiation on circulating blood and immune‐related tissues. Integrating NTCP‐based objectives derived from blood dosimetry parameters—such as mean blood dose or dose to major vessels—would allow the optimizer to directly minimize the predicted risk of radiation‐induced lymphopenia. This biologically informed approach could enable individualized plan optimization that better balances tumor coverage with immune preservation, particularly relevant in the setting of combined radiation and immunotherapy for GBM.

## 5 CONCLUSION

With comparable target coverage, blood vessel sparing during both IMPT and SPArc was found to significantly reduce some blood vessel dose metrics. In addition, SPArc was associated with significantly lower blood vessel dose metrics compared to IMPT, both with and without blood vessel sparing during radiotherapy treatment plan optimization. Blood vessel sparing and clinical adoption of PAT may therefore have the potential to reduce radiation‐induced lymphopenia and improve clinical outcomes.

## AUTHOR CONTRIBUTIONS

**Peilin Liu**: Conceptualization; data curation; formal analysis; investigation; methodology; project administration; validation; writing–original draft; writing–review and editing. **Sebastian Tattenberg**: Conceptualization; data curation; formal analysis; investigation; methodology; project administration; validation; writing–original draft; writing–review and editing. **Xiaoda Cong**: Methodology; writing–review and editing. **Peter Y. Chen**: Writing–review and editing. **Xuanfeng Ding**: Conceptualization; methodology; writing–review and editing. **Abdelkhalek Hamm**: Conceptualization; methodology; writing–review and editing.

## ETHICAL APPROVAL

This study was conducted in accordance with the ethical principles of the Declaration of Helsinki. The study protocol was reviewed and approved by the Institutional Review Board of IRB 2017‐455. The requirement for informed consent to participate was waived by the Institutional Review Board due to the retrospective nature of the study and the use of anonymized data.

## HUMAN ETHICS AND CONSENT TO PARTICIPATE

Not applicable. This study was a retrospective technical and dosimetric analysis using previously generated, de‐identified treatment planning data. No prospective patient enrollment or intervention was performed.

## CONFLICT OF INTEREST STATEMENT

The authors declare no conflicts of interest.

## Supporting information



Supporting Information

## Data Availability

The CT datasets analyzed during the current study are publicly available in the Glioma Image Segmentation for Radiotherapy: RT Targets, Barriers to Cancer Spread, and Organs at Risk (GLIS‐RT) repository (Shusharina N, Bortfeld T, 2021) via The Cancer Imaging Archive: https://doi.org/10.7937/TCIA.T905‐ZQ20. Derived data generated during the current study are available from the corresponding author upon reasonable request.
