# Supplementary material for: Reducing the dose delivered to major intracranial blood vessels in brain radiotherapy using spot‐scanning proton arc therapy
Source: J Appl Clin Med Phys. 2026 Sep 9;27(9):e70780. doi: 10.1002/acm2.70780 (PMC13559178; doi:10.1002/acm2.70780)
Supplement: Supplementary file 1 [file ACM2-27-e70780-s001.docx]

**Supplemental document**

**Table S1.** Registered blood vessel volumes

| Case number | Blood vessel volume (cc) |
| --- | --- |
| 1 | 37.01 |
| 2 | 30.29 |
| 3 | 39.19 |
| 4 | 41.60 |
| 5 | 38.28 |
| 6 | 40.43 |
| 7 | 38.92 |
| 8 | 35.59 |
| 9 | 38.40 |
| 10 | 31.67 |

**Table S2.** Optimization objectives and relative priorities used for treatment planning.

| Structure | Applied to plan | Object function | Relative Priority | Included in robust optimization |
| --- | --- | --- | --- | --- |
| CTV | All plans | D98%>50Gy | 1000 | Yes |
|  | All plans | D0.01%<55Gy | 1000 | Yes |
| Brainstem | All plans | D0.03cc<54Gy | 200 | No |
| Right Optic Nerve | All plans | D0.03cc<54Gy | 200 | No |
| Left Optic Nerve | All plans | D0.03cc<54Gy | 200 | No |
| Optic chiasm | All plans | D0.03cc<54Gy | 200 | No |
| Normal Brain | All plans | D0.03cc<54Gy | 100 | No |
| Blood vessel | Blood vessel–sparing plans only | Dose fall-off from 50Gy to 0Gy over 1cm | 10 | No |

**Table S3.** Beam arrangement of IMPT and SPArc

| Case number | Beam angle/range (°) | |
| --- | --- | --- |
|  | IMPT | SPArc |
| 1 | 30, 120 | 331.3-203.8 |
| 2 | 30, 120 | 341.3-198.8 |
| 3 | 220, 300 | 181.3-338.8 |
| 4 | 30, 180 | 351.3-188.8 |
| 5 | 90, 180 | 351.3-266.3 |
| 6 | 210, 270 | 141.3-36.3 |
| 7 | 0, 90 | 331.3-226.3 |
| 8 | 180, 270 | 131.3-8.8 |
| 9 | 180, 270 | 151.3-8.8 |
| 10 | 180, 270 | 131.3-28.8 |

**Table S4.** Paired statistical comparison of target coverage and conformity index across the four treatment plans.

| **Comparison** | **Median difference (95% CI)** | **r** | **p** | **p (Holm)** |
| --- | --- | --- | --- | --- |
| **Target D98%, Gy(RBE)** | | | | |
| IMPT_Conv_ vs. IMPT_Sparing_ | 0.00 [0.00, 0.01] | 1.00 | 1.000 | 1.000 |
| SPArc_Conv_ vs. SPArc_Sparing_ | 0.00 [0.00, 0.01] | 1.00 | 1.000 | 1.000 |
| IMPT_Conv_ vs. SPArc_Conv_ | 0.00 [0.00, 0.00] | — | — | — |
| IMPT_Sparing_ vs. SPArc_Sparing_ | 0.00 [0.00, 0.00] | — | — | — |
| **Conformity index (CI), unitless** | | | | |
| IMPT_Conv_ vs. IMPT_Sparing_ | −0.050 [−0.075, −0.035] | −1.00 | 0.002 | **0.008 *** |
| SPArc_Conv_ vs. SPArc_Sparing_ | −0.035 [−0.050, −0.005] | −0.82 | 0.020 | 0.059 |
| IMPT_Conv_ vs. SPArc_Conv_ | −0.005 [−0.060, 0.020] | −0.28 | 0.516 | 0.516 |
| IMPT_Sparing_ vs. SPArc_Sparing_ | 0.015 [−0.010, 0.030] | 0.44 | 0.258 | 0.516 |

Median difference = Hodges–Lehmann estimate of the median paired difference with distribution-free 95% confidence interval (95% CI). r = matched-pairs rank-biserial correlation (effect size. p = two-sided exact Wilcoxon signed-rank test; p (Holm) = Holm–Bonferroni-adjusted p across the four pre-specified contrasts within each endpoint. * p (Holm) < 0.05. n = 10 patients. CI in the metric label denotes the conformity index.

**Table S5.** Dosimetric comparison of normal brain and organ-at-risk endpoints across the four planning strategies.

| Dosimetric Parameter | IMPT_Conv_ | IMPT_Sparing_ | SPArc_Conv_ | SPArc_Sparing_ |
| --- | --- | --- | --- | --- |
| Normal Brain_V5Gy(RBE) (%) | 32.76±9.04 | 30.35±8.98 | 34.08±12.1 | 28.23±10.56 |
| Normal Brain V10Gy(RBE) (%) | 27.95±7.91 | 25.6±7.71 | 25.71±7.9 | 21.9±7.18 |
| Normal Brain V20Gy(RBE) (%) | 21.58±5.97 | 19.16±5.43 | 18.53±5.22 | 15.7±4.65 |
| Normal Brain V25Gy(RBE) (%) | 18.78±5.13 | 16.37±4.52 | 16.16±4.47 | 13.56±4.01 |
| Brainstem D_0.03cc_, Gy(RBE) | 37.67±20.14 | 33.77±18.68 | 38.12±20.7 | 32.56±20.47 |
| Right Optic Nerve D_0.03cc_, Gy(RBE) | 14.12±20.61 | 12.14±20.75 | 13.16±20.9 | 11.65±20.51 |
| Left Optic Nerve D_0.03cc_, Gy(RBE) | 16.2±17.82 | 15.03±17.37 | 15.76±18.88 | 11.57±16.35 |
| Optic Chiasm D_0.03cc_, Gy(RBE) | 25.33±21.05 | 20.46±19.53 | 22.93±21.16 | 17.73±21.04 |

VxGy(RBE) (%) denotes the percentage volume of the OAR receiving at least x Gy(RBE). D0.03cc denotes the minimum dose delivered to the hottest 0.03 cm³ of a structure.

**Table S6.** Paired statistical comparison of blood-vessel dose (Dmean and D10%) across the four treatment plans.

| **Comparison** | **Median difference (95% CI)** | **r** | **p** | **p (Holm)** |
| --- | --- | --- | --- | --- |
| **Blood-vessel Dmean, Gy(RBE)** | | | | |
| IMPT_Conv_ vs. IMPT_Sparing_ | 1.09 [0.64, 1.85] | 1.00 | 0.002 | **0.008 *** |
| SPArc_Conv_ vs. SPArc_Sparing_ | 1.68 [1.21, 2.40] | 1.00 | 0.002 | **0.008 *** |
| IMPT_Conv_ vs. SPArc_Conv_ | 0.77 [0.27, 1.74] | 0.89 | 0.010 | **0.010 *** |
| IMPT_Sparing_ vs. SPArc_Sparing_ | 1.31 [0.62, 2.35] | 0.96 | 0.004 | **0.008 *** |
| **Blood-vessel D10%, Gy(RBE)** | | | | |
| IMPT_Conv_ vs. IMPT_Sparing_ | 5.53 [2.68, 10.41] | 1.00 | 0.002 | **0.008 *** |
| SPArc_Conv_ vs. SPArc_Sparing_ | 11.55 [7.75, 15.34] | 1.00 | 0.002 | **0.008 *** |
| IMPT_Conv_ vs. SPArc_Conv_ | 5.32 [2.19, 7.89] | 0.93 | 0.006 | **0.008 *** |
| IMPT_Sparing_ vs. SPArc_Sparing_ | 10.37 [6.48, 13.57] | 0.96 | 0.004 | **0.008 *** |

Median difference = Hodges–Lehmann estimate of the median paired difference with distribution-free 95% confidence interval (95% CI). r = matched-pairs rank-biserial correlation (effect size. p = two-sided exact Wilcoxon signed-rank test; p (Holm) = Holm–Bonferroni-adjusted p across the four pre-specified contrasts within each endpoint. * p (Holm) < 0.05. n = 10 patients.

**Table S7.** Paired statistical comparison of dose-averaged LET (LET_d_) for the target and blood vessels across the four treatment plans.

| **Comparison** | **Median difference (95% CI)** | **r** | **p** | **p (Holm)** |
| --- | --- | --- | --- | --- |
| **Target LET_d_,2%, keV/µm** | | | | |
| IMPT_Conv_ vs. IMPT_Sparing_ | −0.05 [−0.10, 0.00] | −1.00 | 0.125 | 0.375 |
| SPArc_Conv_ vs. SPArc_Sparing_ | −0.15 [−0.25, −0.10] | −0.91 | 0.010 | **0.039 *** |
| IMPT_Conv_ vs. SPArc_Conv_ | 0.15 [−0.15, 0.35] | 0.54 | 0.250 | 0.500 |
| IMPT_Sparing_ vs. SPArc_Sparing_ | 0.10 [−0.35, 0.30] | 0.42 | 0.324 | 0.500 |
| **Target LET_d_,mean, keV/µm** | | | | |
| IMPT_Conv_ vs. IMPT_Sparing_ | 0.00 [−0.05, 0.00] | −1.00 | 0.250 | 0.750 |
| SPArc_Conv_ vs. SPArc_Sparing_ | −0.05 [−0.10, 0.00] | −1.00 | 0.125 | 0.500 |
| IMPT_Conv_ vs. SPArc_Conv_ | 0.00 [−0.10, 0.10] | 0.06 | 1.000 | 1.000 |
| IMPT_Sparing_ vs. SPArc_Sparing_ | 0.00 [−0.15, 0.10] | 0.14 | 0.844 | 1.000 |
| **Blood-vessel LET_d_,2%, keV/µm** | | | | |
| IMPT_Conv_ vs. IMPT_Sparing_ | −0.15 [−0.35, 0.20] | −0.17 | 0.703 | 0.984 |
| SPArc_Conv_ vs. SPArc_Sparing_ | −0.40 [−0.85, 0.40] | −0.51 | 0.203 | 0.813 |
| IMPT_Conv_ vs. SPArc_Conv_ | 1.05 [−1.10, 3.10] | 0.40 | 0.316 | 0.949 |
| IMPT_Sparing_ vs. SPArc_Sparing_ | 0.90 [−1.20, 2.55] | 0.27 | 0.492 | 0.984 |
| **Blood-vessel LET_d_,mean, keV/µm** | | | | |
| IMPT_Conv_ vs. IMPT_Sparing_ | 0.05 [−0.05, 0.30] | 0.52 | 0.313 | 1.000 |
| SPArc_Conv_ vs. SPArc_Sparing_ | −0.05 [−0.15, 0.10] | −0.21 | 0.703 | 1.000 |
| IMPT_Conv_ vs. SPArc_Conv_ | 0.05 [−0.50, 0.95] | 0.07 | 0.871 | 1.000 |
| IMPT_Sparing_ vs. SPArc_Sparing_ | 0.05 [−0.45, 0.70] | 0.02 | 0.980 | 1.000 |

Median difference = Hodges–Lehmann estimate of the median paired difference with distribution-free 95% confidence interval (95% CI). r = matched-pairs rank-biserial correlation (effect size. p = two-sided exact Wilcoxon signed-rank test; p (Holm) = Holm–Bonferroni-adjusted p across the four pre-specified contrasts within each endpoint. * p (Holm) < 0.05. n = 10 patients. LET_d_: dose-averaged linear energy transfer.

**Table S8.** Estimated delivery time comparison

| Case number | IMPT_Conv_ (s) | IMPT_Sparing_ (s) | SPArc_Conv_ (s) | SPArc_Sparing_ (s) |
| --- | --- | --- | --- | --- |
| 1 | 136.49 | 131.94 | 215.8 | 206.98 |
| 2 | 125.81 | 124.62 | 152.18 | 149.16 |
| 3 | 119.59 | 119.36 | 104.34 | 103.71 |
| 4 | 134.97 | 134.55 | 132.96 | 130.66 |
| 5 | 117.75 | 115.7 | 181.02 | 177.42 |
| 6 | 118.51 | 116.29 | 175.46 | 171.62 |
| 7 | 123.62 | 123.41 | 174.77 | 171.25 |
| 8 | 146.56 | 144.65 | 182.79 | 175.64 |
| 9 | 127.44 | 126.27 | 146.81 | 143.73 |
| 10 | 124.48 | 123 | 174.46 | 170.9 |
